# Muscle reorganisation through local injection of stem cells in the diaphragm of *mdx* mice

**DOI:** 10.1186/1751-0147-54-73

**Published:** 2012-12-12

**Authors:** Thais Borges Lessa, Rafael Cardoso Carvalho, André Luis Rezende Franciolli, Lilian Jesus de Oliveira, RodrigoSilvadaNunes Barreto, David Feder, Fabiana Fernandes Bressan, Maria Angélica Miglino, Carlos Eduardo Ambrósio

**Affiliations:** 1 Department of Surgery, School of Veterinary Medicine and Animal Science, University of São Paulo, Cidade Universitária, Avenue: Prof. Dr. Orlando Marques de Paiva, 87, São Paulo, SP, 05508-270, Brazil; 2 Department of Veterinary Medicine, School of Animal Sciences and Food Engineering, University of São Paulo, Avenue: Duque de Caxias Norte, 225, Pirassununga, SP, 13635-900, Brazil; 3 ABC School of Medicine, Avenue: Príncipe de Gales, 821, Santo André, SP, 09060-650, Brazil

**Keywords:** Muscular dystrophy, Mice, Stem cell, Animal model

## Abstract

**Background:**

The diaphragm is the major respiratory muscle affected by Duchenne muscular dystrophy (DMD) and is responsible for causing 80% of deaths. The use of mechanical forces that act on the body or intermittent pressure on the airways improves the quality of life of patients but does not prevent the progression of respiratory failure. Thus, diseases that require tissue repair, such as DMD, represent a group of pathologies that have great potential for cell therapy. The application of stem cells directly into the diaphragm instead of systemic application can reduce cell migration to other affected areas and increase the chances of muscle reorganisation. The *mdx* mouse is a suitable animal model for this research because its diaphragmatic phenotype is similar to human DMD. Therefore, the aim of this study was to assess the potential cell implantation in the diaphragm muscle after the xenotransplantation of stem cells.

**Methods:**

A total of 9 mice, including 3 control BALB/Cmice, 3 5-month-old *mdx* mice without stem cell injections and 3 *mdx* mice injected with stem cells, were used. The animals injected with stem cells underwent laparoscopy so that stem cells from GFP-labelled rabbit olfactory epithelium could be locally injected into the diaphragm muscle. After 8 days, all animals were euthanised, and the diaphragm muscle was dissected and subjected to histological and immunohistochemical analyses.

**Results:**

Both the fresh diaphragm tissue and immunohistochemical analyses showed immunopositive GFP labelling of some of the cells and immunonegativity of myoblast bundles. In the histological analysis, we observed a reduction in the inflammatory infiltrate as well as the presence of a few peripheral nuclei and myoblast bundles.

**Conclusion:**

We were able to implant stem cells into the diaphragm via local injection, which promoted moderate muscle reorganisation. The presence of myoblast bundles cannot be attributed to stem cell incorporation because there was no immunopositive labelling in this structure. It is believed that the formation of the bundles may have been stimulated by cellular signalling mechanisms that have not yet been elucidated.

## Background

Duchenne muscular dystrophy (DMD) has been described as one of the most devastating, rapidly progressive and severe forms of hereditary myopathies, with an incidence of 1 in 3,500 males born [1,2]. DMD is an X-linked recessive disease caused by a mutation in the gene encoding a 427-kDa protein located on the short arm of chromosome X at locus Xp21 [3].

Dystrophin represents only 0.002% of the striated muscle cell mass and is located at the intracellular surface of the sarcolemma in combination with several integral membrane glycoproteins, forming the dystrophin-associated glycoprotein complex (DGC). The DGC is responsible for the membrane permeability of muscle cells that promotes the binding of F-actin, a thin myofilament protein, to dystroglycan, forming the DGC of cardiac and skeletal striated muscle cells and smooth muscle cells [4]. Although the function of the DGC has not been elucidated in the literature, it may have a role in cytoskeleton structural integrity and cell survival signalling [5].

The first symptoms of DMD are usually reported at 2 years of age, but parents only recognise them at approximately age 5, when the child begins to have difficulties going up or down stairs and falls [6]. At approximately 20 to 30 years of age, depending on the ventilation resources used, the patients die as a result of respiratory muscle impairment, which is the cause of death in 80% of DMD patients, due to respiratory failure in addition to an infection or heart failure. Structural deformities are characteristic of DMD patients, resulting from their confinement to a wheelchair. The most serious structural deformity is scoliosis, the main culprit underlying the drastic reduction in lung function. Due weakening of respiratory muscle, these patients are unable to generate respiratory pressure (maximal inspiratory pressure and maximal expiratory pressure), and the peak expiratory flow is reduced. Thus, all of these factors lead to the premature death of DMD patients [3,7,8]. The main failure of muscles involved in breathing occurs in the diaphragm. The reduction in lung compliance and chest wall mobility leads to an increased mechanical load sustained with each breath. The onset of hypercapnia, pulmonary hypoventilation, hypoxaemia (following hypercapnia) and, consequently, the clearance of secretions results from weakened musculature. The uses of mechanical forces acting on the body or intermittent pressures acting on the airways have helped to improve the performance of inspiratory and expiratory muscles. Among them, negative pressure body ventilators (NPBVs) and bilevel positive airway pressure (BiPAP) improve the quality of life of patients but do not prevent the progression of respiratory failure [6].

Similarly to human DMD, in *mdx* mice (X chromosome-linked muscular dystrophy in the mouse) muscle inflammation begins at 3 weeks of age, peaking between 8 and 12 weeks. After this period, inflammation disappears spontaneously from the muscles. However, the *mdx* diaphragm muscle shows moderate endomysial fibrosis that is aggravated by intense oxidative stress at 3 months of age and worsens at 6 months of age without the clearance of inflammation [9]. This disease progression is similar to human DMD [10,11].

The life span of the *mdx* mouse is reduced, and respiratory and/or cardiac insufficiency is the main cause of death [12,13]. Recent studies performed using whole-body plethysmography have shown that the respiratory rate, tidal volume and minute volume are significantly reduced in the *mdx* animals from 2 to 6 months of age and may worsen at 7 months of age [13,14]. In contrast, some less recent data report that there is only a small difference in the tidal volume (Vt) in response to hypercapnia in *mdx* mice at 7 months and little ventilation in *mdx* mice at 5 months of age [13,15].

Diseases that require tissue repair, including DMD, represent a group of pathologies that have great potential for cell therapy. Currently, the use of stem cells has expanded into new areas of biotechnology and has been beneficial to the field of regenerative medicine [16].

Mesenchymal stem cells (MSCs) are characterised as adherent fibroblastoid cells. They have the capacity to differentiate into connective tissue cells, including adipocytes, osteocytes and myocytes, and there is evidence that MSCs can selectively differentiate in injured tissues [17]. Among the stem cells source in adult organisms, the bulb olfactory has been studied as a potential source of multipotent stem cells in many species. For example, in rats, the fibroblast-like cells isolated from the olfactory bulb showed to express mesenchymal cells markers such as CD29 and CD90 and are also able to differentiate along osteoblastic, adipogenic and chondrogenic lineages [18].

Neural stem cells can be characterized on a critical functional basis in terms of their undifferentiated features, capacity for self-renewal, pluripotentiality, and ability to regenerate damaged tissue and these characteristics have been found in culture of embryonic and adult murine brain [19]. One of the most promising sources of neural stem cells with unique characteristics are the olfactory epithelial stem cells because they are close to the higher central nervous system (CNS) and may be easily obtained in humans through a biopsy of the external nostrils [20].

Therefore, the aimed to assess the potential cell implantation and muscle morphology following xenotransplantation performed by the local injection of stem cells into the dystrophic diaphragm.

## Materials and methods

This research was certified by the Ethical Principles in Animal Research adopted by “Ethic Committee in the use of animals” of the School of Veterinary Medicine and Animal Science of University of São Paulo, protocol number 2045/2010.

### Animals

A total of 6 *mdx* mice from the Vivarium at FMABC and 3 BALB/C57 mice from the Pathology Vivarium at FMVZ/USP were used. All animals used in the experiment were 5-month-old males. The animals were divided into the following 3 groups:

Group A 3 BALB/C57 control mice without mesenchymal stem cell implantation;

Group B 3 *mdx* controls without mesenchymal stem cell implantation;

Group C 3 *mdx* mice treated with local injections of mesenchymal stem cells.

### Isolation, culture and genetic modification of adult stem cells

Mesenchymal stem cells have been previously isolated from rabbit olfactory epithelium (REF), cultured in DMEM/F12 medium supplemented with 10% foetal bovine serum, 1% nonessential amino acids, glutamine and antibiotics and characterised (Ambrosio et al., unpublished data). The cells were exposed to lentiviral transduction for enhanced green fluorescent protein (eGFP) expression [21,22]. Briefly, the viral particles were produced in 293FT cells via lipofection of packaging plasmids and the FUGW plasmid. After 72 h of incubation, the supernatant was filtered at 0.45 μm and used for the infection of 2 x 10^5^ cells for approximately 16 h. After this period, the culture medium was replaced, and the cells were cultured *in vitro* for a minimum of 3 days before use *in vivo*.

### Laparoscopy

The laparoscopic procedure was performed at the Veterinary Hospital of FMVZ-USP. Anaesthesia was initiated intraperitoneally with a combination of 50 mg/kg ketamine hydrochloride (Ketamin-S®, Cristália) and 2 mg/kg xylazine hydrochloride (Calmiun®, Agener União), and the animals were kept under spontaneous breathing.

The animals were positioned in dorsal decubitus with a 20-degree elevation of the forelimbs. Subsequently, an incision was made at the midline sagittal plane under the 0.5-cm linea alba in the lower third of the abdomen to introduce the 2.7-mm laparoscope. The laparoscope was fixed with an “X” suture, and the pneumoperitoneum was established using a 25X7G needle parallel to the laparoscope with carbon dioxide (CO_2_). The surgery proceeded, and upon visualisation of the diaphragm muscle, a 24G catheter needle connected to an insulin syringe containing the MSCs was introduced into the 10th intercostal space.

### Xenotransplantation

Mesenchymal stem cells from rabbit olfactory epithelium were xenotransplanted at a concentration of 2x10^5^ cells suspended in sterile saline solution. No immunosuppressive drugs were used.

### Prophylactic protocol

The post-surgical protocol consisted of 2.5 mg/kg flunixin meglumine for 3 days and 2 mg/kg of meperidine hydrochloride 3 times a day for 5 days for analgesia. For prophylactic antibiotic therapy, 10 mg/kg enrofloxacin was administered intramuscularly twice per day. After 8 days, the animals were euthanised with an overdose of anaesthesia, and the diaphragmatic muscles, together with the intercostal muscles, were collected in sterile plates containing PBS solution to wash the material.

### Histological analysis

In all groups, the diaphragm muscle fragments were collected and fixed in 4% paraformaldehyde for 24 hours. They were then dehydrated in an ascending ethanol series (70% to 100%), made diaphanous in xylene and embedded in Paraplast® (Leica/Germany), from which 3x4 cm rectangular blocks were prepared. Then, 5-μm serial sections were generated using an automatic microtome (Leica RM 2065) to prepare the slides, which were subsequently deparaffinised in an oven at 60°C for 2 hours.

Hematoxylin-eosin staining was used for the structural and histopathological analysis of the dystrophic muscle characteristics [23]. The slides were imaged using an Olympus BX 60 Microscope coupled to an Axio CAM HRc camera with Zeiss® KS 400 software.

### Immunohistochemical analysis

The identification of GFP (green fluorescent protein) in the diaphragm of the animals euthanised 8 days after the injection of rabbit olfactory epithelial stem cells was performed using anti-GFP antibodies (Living Colours GFP Monoclonal Antibody, Clontech, cat. # 632375, Foster City,Califórnia 94404, USA).

The 5-μm sections were deparaffinised in xylene and rehydrated in a series of solutions with decreasing ethanol concentrations. The sections were heated in citrate buffer (0.384 g citric acid monohydrate and 2.352 g sodium citrate tribasic dihydrate in 1 L of distilled water, pH 6.0) for 15 minutes in a microwave oven to perform antigen retrieval. The endogenous tissue peroxidase activity was blocked by incubation in a 3% solution of hydrogen peroxide in 1 M Tris–HCl buffer, pH 7.5 (TBS, 60.57 g Tris in 500 mL ultrapure water), for 30 minutes. The sections were incubated with 10% goat serum in TBS for 30 minutes to block non-specific binding. The primary antibody was diluted to 0.2 mg/mL in TBS buffer containing 1% goat serum and incubated overnight at 4°C in a moist chamber. In parallel, slices were incubated at the same concentration of irrelevant antibody as an isotype control (IgG, anti-mouse IgG).

After incubation with the primary antibody, all sections were washed with TBS containing 1% goat serum. The reaction was visualised using the multipurpose Dako Advance HRP Link kit (cat. # K4069, Dako, USA) according to the manufacturer’s instructions. The reaction was visualised by precipitation of 3,3′-diaminobenzidine (DAB Peroxidase Substrate Kit, 3,3′-diaminobenzidine, cat. # SK-4100). Finally, the sections were counter-stained with hematoxylin, dehydrated and mounted on slides for light microscopy analysis.

## Results

### Structural analysis of the diaphragm muscle

**Figure 1:**
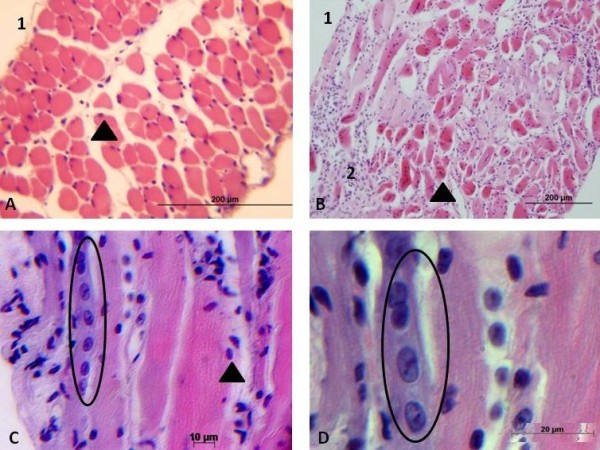
**Photomicrograph of mdx diaphragm muscle before and after stem cells transplantation.** In (**A**) normal BALB/C57 mouse, showing epimysium (1) and basal nuclei (arrow) in the muscle fibers; (**B**) Control mdx mouse, showing epimysium (1), perimysial fibrosis (2) and the central nucleus (arrow).In (**C**) mdx stem cell treated group, (arrow) indicates basal nuclei and myoblast bundle in the circle. (**D**) high magnification of myoblast bundle in the circle.

In the structural analysis of the diaphragm in the control animals, group A (Figure 1, A), the presence of basal nuclei and epimysium was observed. We found that the diameter of the muscle fibres was preserved in this group of animals, and there was no inflammatory infiltrate, according to the normal muscle morphology [2].

In the *mdx* group that not received stem cell implants, i.e., group B (Figure 1, B), the epimysium is present and a robust perimysial inflammatory infiltrate was observed. Moreover, the muscle cells displayed central nuclei. In these animals, we observed the presence of fibres with various diameters.

Some basal nuclei can be observed in mdx stem cell treated group (Figure 1, C).The presence of a myoblast bundle is noted only at stem cell treated group and exhibit long and tubular appearance can be observed in low (Figure 1, C) and high magnification (Figure 1, D).

### Cell implantation analysis

**Figure 2:**
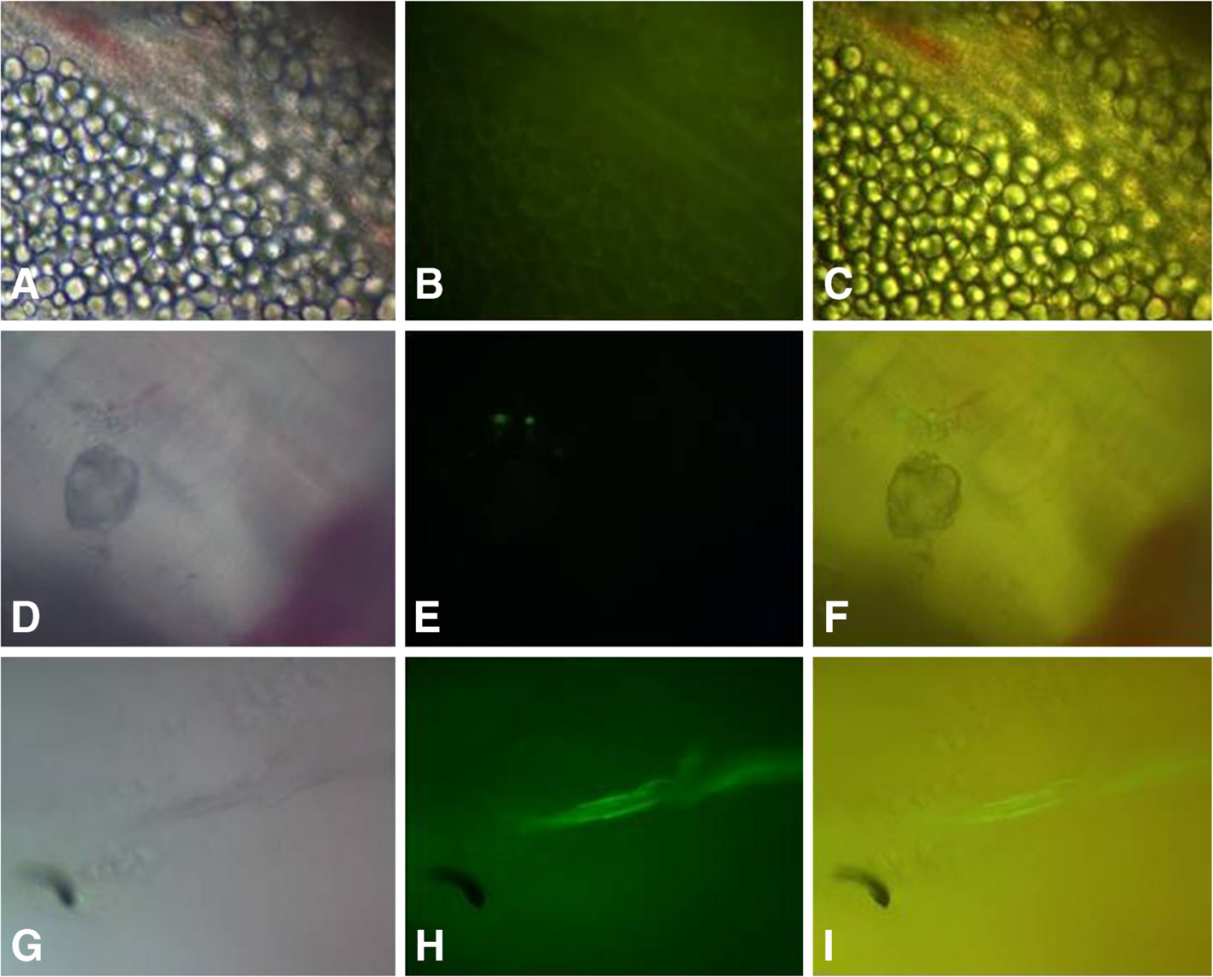
**Analysis of the diaphragm and intercostal muscle from mdx with stem cell Implant by whole**- **mount epifluorescence microscopy.** GFP-positive staining can be observed in the diaphragm (**A**, **B** and **C**); (**D**, **E** and **F**) are niches of GFP-labelled stem cells. The fluorescent intercostal muscles are noted in (**G**, **H** and **I**), tracing a linear path.

In the fresh tissue analysis of the diaphragm using an inverted fluorescence microscope, it was possible to identify clusters of GFP-positive stem cells located throughout the diaphragm. In the intercostal muscles, there was a linear structure adjacent to the path of the needle showing the injection of stem cells, which is represented in the image by a fluorescent structure (Figure 2).

**Figure 3:**
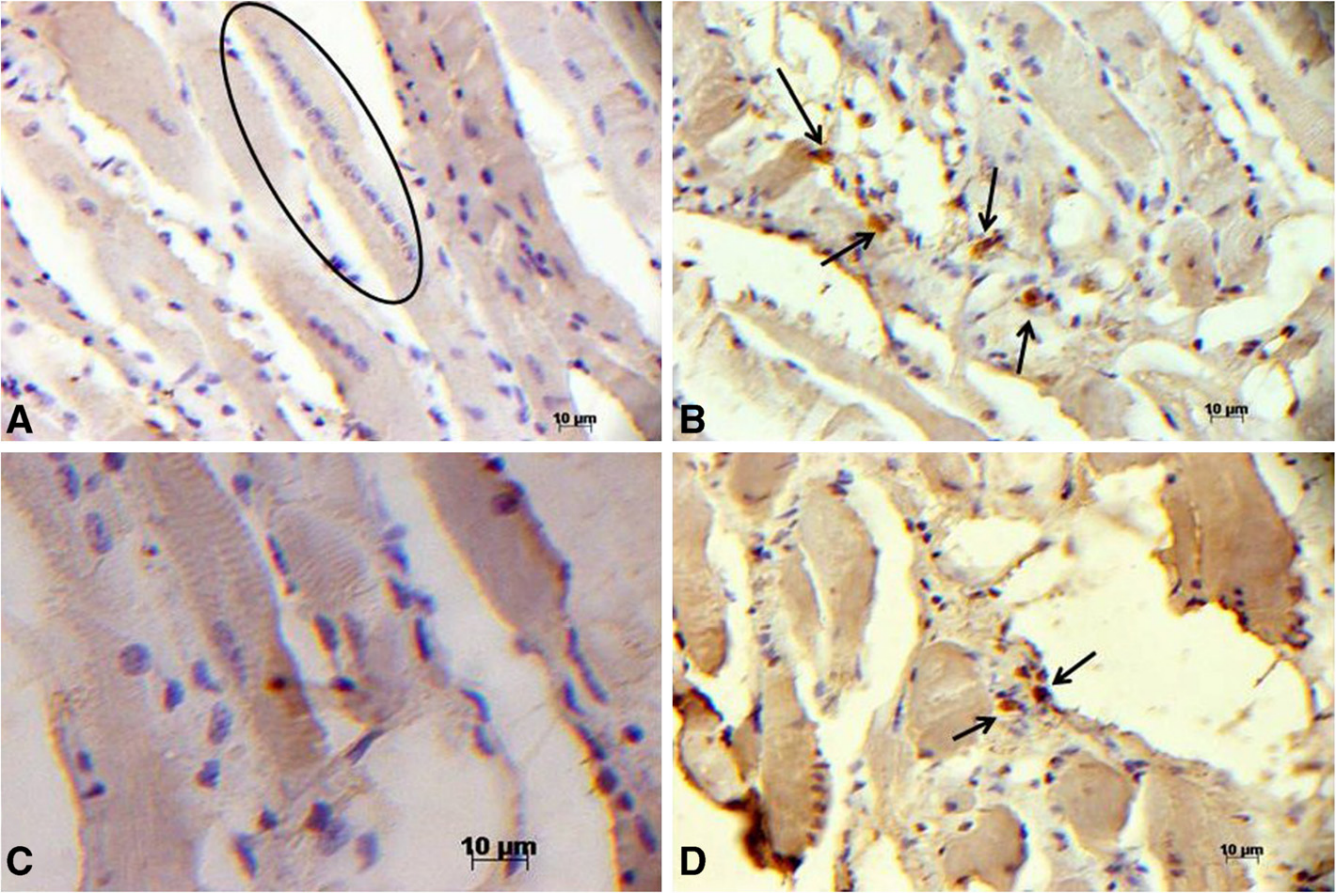
**GFP detection by immunohistochemistry in longitudinal sections of the diaphragm muscle.** (**A**) An *mdx* diaphragm transplanted with stem cells; within the circle, there is an immunonegative string of myoblasts; (**B** and **D**) *mdx* diaphragm transplanted with stem cells, GFP-immunopositive labelling (→) and (**C**) the rectus abdominis muscle of an *mdx* mouse without a stem cell implant as an immunonegative control.

In the immunohistochemical reaction with the anti-GFP antibody, no labelling of the strings of myoblasts or muscle fibres was observed. Immunopositive labelling was observed in some cells with dense nuclei present in the perimysial fibrosis, indicating that the injection enabled the implantation of stem cells (Figure 3). These findings confirm the described morphological results using fresh tissue immunofluorescence.

## Discussion

The *mdx* mouse is an animal model that is amenable to preclinical trials regarding changes in the diaphragm muscle. This muscle exhibits morphology, biochemistry and functions similar to human DMD and may contribute to new studies of diseases involving the diaphragm.

Currently, regenerative medicine has been applied to several cases, including myocardial infarction [24], pulmonary diseases [25], tendinitis [26] and muscle dysfunction [27]. By applying regenerative medicine to the aforementioned regenerative diseases, the current study contributes to the use of a safe cell therapy technique.

The *mdx* mouse has been used as an experimental model in procedures involving the use of stem cells (Gussoni et al., 1999 [28]; McKinney-Freeman et al., 2002 [29]; Corbel et al. [30]; Fukada et al., 2001[31]; Hagiwara et al., 2006 [32]), showing that this model has been useful for DMD studies.

To best of our knowledge this is the first report on stem cells local level transplantation in the diaphragm of *mdx* mouse. This strategy reduces the migration of stem cells to other affected areas and to increase the chances of muscle reorganisation.

The use of cell therapy in the diaphragm muscle was previously describe by Zhang et al., 2006 [33]. They examined the effects of xenotransplantation of bone marrow-derived stem cells isolated from normal rats into the diaphragm muscles of mdx mice by injection into the caudal vein in 18 8-week-old *mdx* females. These animals were euthanised at 4, 8 and 12 weeks after the transplantation of stem cells. The authors found that the proportion of centrally nucleated muscle fibres was slightly altered compared to the group that received no stem cell injection, and there was a reduction in the inflammatory infiltrate.

Regarding the methodology used to access the diaphragm, the current study has proposed an alternative that is easy to perform and safe. In contrast to the study by Zhang et al., 2006 [33], we used male animals with local laparoscopic application of stem cells in a specific region of the diaphragm (costal plane). This method was proven effective given the histological and immunohistochemical findings described. These results lead us to believe that both the systemic and local use of cells may provide the same efficacy regarding diaphragm muscle reorganisation.

Another issue that should be discussed is the type of cells used for such therapy. Several authors [28,29,31,32], used in mdx mouse, bone marrow cells for DMD treat. Zhang et al., 2006 [33] contributed the use of fetal liver cells and observed the same results as those obtained when using bone marrow.

Few studies have reported the use of rabbit olfactory epithelial stem cells. Marshall et al., 2006 [34] suggested that olfactory epithelial stem cells show a putative potential for axonal regeneration and for application in demyelinating diseases. During a 3-year clinical trial in patients with chronic spinal cord injury, Mackay-Sim et al., 2008 [35] used autologous transplantation of olfactory epithelial cells in humans and found no clinical changes or tumour formation after transplantation.

Currently, several researchers have been used cell therapy with cells from olfactory epithelium to in order to recovery of spinal cord injuries (Raisman, 2007; Ramer, et al., 2004; Li et al., 2004; Ramón-Cueto et al., 1998). In none of these authors are cited the use of immunosuppressive pre or post treatment, as well as immune response is not reported rejection. Furthermore, there are reports indicating that the use of immunosuppressant agents can improve the muscular function by itself (ref) that would affect the observation of the effects of stem cells transplant in our model.

In the present study, we used rabbit olfactory epithelial cells, which were shown to be potentially effective in reorganising diaphragm muscles, given that myoblast bundle formation was clear. However, we believe that this finding resulted from cellular signalling events that remain unknown to this date; thus, we cannot attribute this event to the cell type used.

## Conclusion

Although the *mdx* mouse diaphragm muscle is a membranous structure and, therefore, presents difficulties in direct intervention, it was shown that stem cell implantation via local application to this muscle generated moderate muscle reorganisation. The diaphragm muscle exhibited decreased inflammatory infiltrate 8 days after xenotransplantation. In addition, basal nuclei and the myoblast bundle were observed, confirming stem cell implantation in the muscle. We believe that this experiment opens a new avenue for future applications and cell therapy in the dystrophic dog model, once it can helps ameliorate the respiratory symptoms associated to muscle dystrophy disease.

## Abbreviations

DMD: Duchenne muscular dystrophy; mdx: X chromosome-linked muscular dystrophy in the mouse; GFP: Green fluorescent protein.

## Competing interests

The authors declare that they have no competing interests.

## Authors’ contributions

TBL, RCC, ALRF, RSNB, LJO, FFB and CEA performed the experiments and wrote the manuscript. TBL, DF, FFB, MAM and CEA participated in the design of the study, coordination and helped wrote the manuscript. All authors read and approved the final manuscript.
